# The impact of autopsy participation on clinical residency

**DOI:** 10.1002/jgf2.449

**Published:** 2021-05-03

**Authors:** Kohta Katayama, Yuji Nishizaki, Tomohiro Shinozaki, Yuta Saitoh, Tetsuhiro Yano, Takuya Aoki, Masayuki Noguchi, Yasuharu Tokuda

**Affiliations:** ^1^ Department of General Medicine Shirakawa Satellite for Teaching And Research (STAR) Fukushima Medical University Fukushima Japan; ^2^ Department of Medical Education Juntendo University School of Medicine Tokyo Japan; ^3^ Department of Information and Computer Technology Faculty of Engineering Tokyo University of Science Tokyo Japan; ^4^ Department of Internal, Emergency, and General Medicine Saitama Citizens Medical Center Saitama Japan; ^5^ Department of Emergency Medicine Fukushima Medical University Fukushima Japan; ^6^ Division of Clinical Epidemiology The Jikei University School of Medicine Tokyo Japan; ^7^ Department of Pathology Faculty of Medicine University of Tsukuba Tsukuba City Japan; ^8^ Muribushi Okinawa Center for Teaching Hospitals Urasoe City Japan

**Keywords:** autopsy, clinical residency, clinicopathological conference, Japan

## Abstract

**Background:**

Autopsy has had an essential role in ensuring the quality of education and medical care. However, its role in clinical residency has not been clarified. This study assessed actual autopsy circumstances during clinical residency and evaluated the association between autopsy and clinical knowledge.

**Methods:**

We conducted a cross‐sectional study involving postgraduate second year residents in Japan who took the General Medicine In‐Training Examination in 2019. We modeled the General Medicine In‐Training Examination scores of the residents to examine their association with autopsy experiences and the number of autopsy experiences to assess its predictors.

**Results:**

Of 2715 postgraduate second year residents, 353 (13.8%) had no autopsy participation, and 1015 (39.7%) had only one experience. Although autopsy participation was not related to the mean General Medicine In‐Training Examination score, the residents' clinicopathological conference participation, self‐study for more than 60 min per day, and wish to be pathologists were significantly associated with autopsy experiences. They experienced more autopsies when they belonged to small‐sized hospitals in rural areas performing many autopsies.

**Conclusion:**

We reported the current status of autopsy in clinical residency and showed that more than half of the residents experienced no or only one autopsy. General Medicine In‐Training Examination scores were not correlated with the number of autopsy experiences.

## Graphical abstract

We reported that more than half of resident physicians experienced no or only 1 autopsy. Although General Medicine In‐Training Examination scores were not correlated with the number of autopsy experiences, highly motivated residents in a rural area experienced more autopsies.

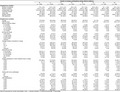

## 1 INTRODUCTION

Historically, autopsy has made an invaluable contribution to medicine, from understanding novel illnesses to answering patient management issues and maintaining quality of medicine. The value of autopsy in detecting diagnostic errors has been demonstrated.^1^, ^2^, ^3^, ^4^, ^5^ In a retrospective review of 2 year records from an educational hospital, 34% of autopsy cases had an unexpected pathological diagnosis leading to death, and 93% of the physicians who attended the autopsies rated them as being a valuable educational experience.^6^ However, as a global trend, the autopsy rate has been continuously decreasing.^7^ In Japan, it was 5.2% in 2012, and an autopsy was performed in approximately half of all in‐hospital deaths in the 1960s.^8^

In Japan, residents were required to participate in autopsies and clinicopathological conferences (CPCs) since the new postgraduate medical education (PGME) program was introduced in 2004.^9^ Residents must attend the autopsy explanation to the bereaved families, the autopsy itself, and CPCs to understand the pathophysiology in detail during their 2 years of clinical residency training. The purpose of PGME is training physicians with a holistic approach and acquiring primary care skills. This training, from the autopsy explanation to CPCs, is considered a part of this holistic approach. In a survey for each teaching hospital conducted jointly by the Japanese Society of Pathology and the Japanese Society of Internal Medicine, residents learned autopsy explanation, including its permission, by observing their supervising physicians.^10^ In addition, the survey has found that autopsy education has become an on‐the‐job training. How many autopsies residents experience and how effective autopsies are in the PGME program are unclear.

During these 16 years, we have verified the new PGME program using the General Medicine In‐Training Examination (GM‐ITE), which was developed using a similar methodology for developing the Internal Medicine ITE (IM‐ITE) in the United States.^11^ The purpose of the GM‐ITE (the same as that of the IM‐ITE) is providing residents and program directors with an objective, reliable, and valid assessment of each resident's clinical knowledge in a multiple‐choice examination, and the mean scores of each program were compared with those of their peers.^12^, ^13^ We have been examining the characteristics of residents regarding their clinical knowledge using a questionnaire survey conducted at the same time as the GM‐ITE. In our previous studies, we have reported the characteristics of residents with more excellent clinical knowledge, such as those with appropriate emergency department and inpatient caseloads and those working in provincial community hospitals with many beds.^14^, ^15^, ^16^, ^17^

We hypothesized that autopsy would be one of the factors associated with clinical knowledge because autopsy has become a compulsory part of PGME. Therefore, we used the GM‐ITE and a concurrent questionnaire survey to clarify the actual circumstances of autopsy among residents and evaluated the association between their autopsy experiences and clinical knowledge.

## 2 METHODS

### 2.1 Study design and study population

We conducted a cross‐sectional study involving postgraduate second year (PGY‐2) residents of 441 teaching hospitals in Japan who took the GM‐ITE in 2019. The participants were trainees of 2 year postgraduate rotation training programs, including internal and emergency medicine, required for all residents regardless of their specialty before entering the specialty training programs (PGY‐3 or later). All participants provided informed consent, which was obtained under the opt‐out agreement. The residency program directors were required to assemble residents in a room at each hospital at a scheduled time and administer the GM‐ITE to their residents. Then, each program director collected the completed examination answer sheets and sent them back to us in an envelope we provided. Since the academic calendar in Japan starts on April 1 and ends on March 31 of the following year, the GM‐ITE was conducted in February or March 2019. Immediately after the test, we provided a self‐reported questionnaire sheet regarding the residents' autopsy experiences (i.e., the number of autopsies experienced, autopsy participants when they were in charge, and CPC participation). In addition, the sheet included the question whether residents wished to be pathologists.

We collected the number of autopsy cases and deaths at each hospital from the Annual of the Pathological Autopsy Cases in Japan by the Japanese Society of Pathology. This database has registered all autopsies performed in Japan since 1960. We used the data from 2017 (11,089 cases registered from 808 hospitals). In addition, we obtained additional data, including whether it was a university hospital, whether it was located in an urban area, how many beds it had, and whether it had a general medicine department, from the website of each hospital.

This study was approved by the Institutional Review Board of Mito Kyodo General Hospital, Mito City, Ibaraki, Japan.

### 2.2 General Medicine In‐Training Examination

In Japan, since the new PGME program was introduced in 2004, the management and implementation of the training program were left primarily to the discretion of each teaching hospital. Furthermore, objective outcome indicators in clinical training have not been established, and the training contents during residency varied depending on each hospital. In September 2005, the Japan Organization of Advancing Medical Education Program (JAMEP), a nonprofit organization, was established to resolve these issues. The JAMEP developed the GM‐ITE as an objective evaluation indicator of the resident's basic clinical knowledge. The GM‐ITE, an “in‐training examination” for residents, was introduced in 2012 (first edition), and the number of participants has increased every year. In 2019 (eighth edition), 6133 residents from 503 teaching hospitals took the GM‐ITE.

The GM‐ITE included 60 questions testing a wide range of clinical knowledge, from clinical skills and practical medical knowledge to patient psychosocial care. The examination was designed and written by a committee of experienced attending physicians organized by the JAMEP. Questionnaires presented at the GM‐ITE focused on residents' practical experience, not just questions on their knowledge. The maximum and minimum scores for the examination were 60 and 0, respectively, with higher scores indicating a better performance of the general medicine knowledge base. Before conducting the examination, a question review was organized, and the content validity was confirmed by the peer review of each committee member.

### 2.3 Statistical analyses

We summarized the residents' (resident‐level variables) and workplace (hospital‐level variables) characteristics using the self‐reported number of autopsy participation during their clinical residency programs. The purposes of our analysis were as follows: (a) to assess the association between GM‐ITE scores and residents' autopsy experiences after adjusting the characteristics and (b) to investigate the possible characteristics associated with the residents' autopsy experiences. For the first purpose, we fitted the mixed‐effect models for the GM‐ITE scores by incorporating hospital variation as normally distributed random intercepts. The numbers of autopsies and deaths at each hospital were adjusted separately in Model 1, whereas the ratio of these numbers and resident‐ and hospital‐level characteristics were adjusted in Model 2. For the second purpose, the mean number of autopsy experiences was modeled using linear and log‐linear models through the generalized estimating equations, treating hospitals as clusters with the independence working correlation. The linear and log‐linear models estimated the increase in the average number of autopsy experiences by each variable on the additive and multiplicative scales, respectively.

However, note that because the highest category of the number of autopsy experiences was censored seven times (i.e., outcome values range from 0 to 7), estimates may include the bias from the “ceiling effect.” Hence, we conducted sensitivity analyses. First, we excluded residents who participated in more than seven autopsies (*n* = 36). Second, the number of autopsy participation was censored using the discrete‐time hazard model (with the robust variance estimator clustering hospitals). The model was interpretable as a continuation‐ratio logit model for ordinal categories (i.e., the numbers of autopsy experiences) and provided estimates of common odds ratios for the probability of taking several outcomes (autopsy participation) among the residents with more than or equal to that number. Tables S1 and S2 show the results of the sensitivity analyses.

All analyses were conducted through SAS version 9.4 (SAS, Inc.).

## 3 RESULTS

A summary of the baseline characteristics is shown in Table 1. In total, 2715 PGY‐2 residents took the GM‐ITE in 2019. Among them, we retrospectively analyzed 2554 residents from 441 hospitals (response rate was 94.1%). Of these participants, 47 (1.7%) wished to be pathologists. We obtained all hospital‐level variables from the Annual of the Pathological Autopsy Cases in Japan by the Japanese Society of Pathology and the websites of each hospital. The average GM‐ITE score was 31.6 ± 6.0. During the 2 year clinical residency, 353 (13.8%) residents had no autopsy experience, 1015 (39.7%) had one autopsy experience, 608 (23.8%) had two autopsy experiences, 337 (13.2%) had three autopsy experiences, 112 (4.4%) had four autopsy experiences, 67 (2.62%) had five autopsy experiences, 26 (1.0%) had six autopsy experiences, and 36 (1.4%) had more than seven autopsy experiences. The corresponding number of autopsy experiences of which the residents were in charge were 1307 (51.2%), 987 (36.7%), 195 (7.6%), 42 (1.7%), 13 (0.5%), four (0.2%), two (0.1%), and two (0.1%), respectively.

**TABLE 1:** Factors related to GM‐ITE score compared with number of autopsy experiences during clinical residency

	Number of autopsy participations during clinical residency							
	0	1	2	3	4	5	6	>7
	(*n* = 353)	(*n* = 1015)	(*n* = 608)	(*n* = 337)	(*n* = 112)	(*n* = 67)	(*n* = 26)	(*n* = 36)
Hospital‐level variables								
Number of autopsies	17.8 ± 14.9	15.4 ± 13.8	14.4 ± 14.0	18.2 ± 18.7	19.7 ± 19.4	20.3 ± 25.3	16.1 ± 18.8	14.6 ± 18.3
Number of deaths	473.4 ± 273.2	436.3 ± 242.5	396.6 ± 224.3	433.7 ± 221.9	424.9 ± 198.2	385.8 ± 223.8	380.4 ± 199.0	389.3 ± 200.6
Autopsy/death ratio	0.03 [0.02, 0.05]	0.03 [0.02, 0.04]	0.03 [0.02, 0.04]	0.03 [0.02, 0.05]	0.04 [0.02, 0.06]	0.04 [0.02, 0.07]	0.03 [0.01, 0.06]	0.03 [0.02, 0.05]
University Hospital	66 (18.7)	103 (10.2)	55 (9.1)	35 (10.4)	20 (17.9)	10 (14.9)	4 (15.4)	2 (5.6)
Urban area	144 (40.8)	364 (35.9)	186 (30.6)	101 (30.0)	28 (25.0)	13 (19.4)	8 (30.8)	13 (36.1)
Number of beds	655.2 ± 258.0	557.5 ± 215.5	519.9 ± 188.2	545.3 ± 199.1	546.9 ± 220.2	513.4 ± 210.2	479.8 ± 167.5	463.6 ± 189.2
GM department	238 (67.4)	585 (57.6)	341 (56.1)	196 (58.2)	65 (58.0)	37 (55.2)	20 (76.9)	13 (36.1)
Resident‐level variables								
Female	115 (32.6)	353 (34.8)	188 (30.9)	129 (38.3)	38 (33.9)	8 (11.9)	12 (46.2)	9 (25.0)
GM‐ITE score	31.5 ± 6.1	31.6 ± 5.8	31.5 ± 5.9	32.6 ± 6.4	32.3 ± 5.9	32.6 ± 5.7	32.5 ± 6.0	30.2 ± 5.5
Internal medicine rotation								
0–5 months	27 (7.7)	42 (4.1)	30 (4.9)	12 (3.6)	4 (3.6)	2 (3.0)	2 (7.7)	2 (5.6)
6–10 months	233 (66.2)	710 (70.0)	415 (68.4)	223 (66.2)	78 (69.6)	50 (74.6)	17 (65.4)	26 (72.2)
11–15 months	77 (21.9)	234 (23.1)	144 (23.7)	92 (27.3)	27 (24.1)	15 (22.4)	7 (26.9)	7 (19.4)
16–20 months	13 (3.7)	25 (2.5)	15 (2.5)	8 (2.4)	3 (2.7)	0 (0)	0 (0)	1 (2.8)
>21 months	2 (0.6)	4 (0.4)	3 (0.5)	2 (0.6)	0 (0)	0 (0)	0 (0)	0 (0)
ED duty per month								
0 per month	10 (2.8)	27 (2.7)	11 (1.8)	8 (2.4)	4 (3.6)	2 (3.0)	1 (3.9)	4 (11.1)
1–2 per month	64 (18.1)	144 (14.2)	75 (12.3)	28 (8.3)	13 (11.6)	7 (10.5)	6 (23.1)	23 (63.9)
3–5 per month	230 (65.2)	723 (71.2)	423 (69.6)	258 (76.6)	67 (59.8)	48 (71.6)	16 (61.5)	9 (25.0)
>6 per month	47 (13.3)	110 (10.8)	90 (14.8)	40 (11.9)	27 (24.1)	10 (14.9)	3 (11.5)	0 (0)
Unknown	2 (0.6)	11 (1.1)	9 (1.5)	3 (0.9)	1 (0.9)	0 (0)	0 (0)	0 (0)
Average number of inpatients in charge								
0–4	77 (21.8)	139 (13.7)	91 (15.0)	49 (14.6)	20 (17.9)	9 (13.4)	3 (11.5)	7 (19.4)
5–9	202 (57.2)	599 (59.1)	348 (57.3)	189 (56.4)	67 (59.8)	44 (65.7)	15 (57.7)	20 (55.6)
10–14	43 (12.2)	184 (18.2)	111 (18.3)	53 (15.8)	15 (13.4)	11 (16.4)	6 (23.1)	8 (22.2)
>15	20 (5.7)	59 (5.8)	41 (6.8)	26 (7.8)	7 (6.3)	1 (1.5)	2 (7.7)	1 (2.8)
Unknown	11 (3.1)	33 (3.3)	16 (2.6)	18 (5.4)	3 (2.7)	2 (3.0)	0 (0)	0 (0)
Study time per day								
0–30 min	145 (41.1)	338 (33.3)	192 (31.6)	100 (29.8)	41 (36.6)	25 (37.3)	2 (7.7)	8 (22.2)
31–60 min	135 (38.2)	417 (41.1)	244 (40.1)	152 (45.2)	31 (27.7)	24 (35.8)	12 (46.2)	10 (27.8)
61–90 min	46 (13.0)	149 (14.7)	115 (18.9)	53 (15.8)	32 (28.6)	12 (17.9)	8 (30.8)	7 (19.4)
>91 min	10 (2.8)	48 (4.7)	31 (5.1)	11 (3.3)	3 (2.7)	3 (4.5)	1 (3.9)	8 (22.2)
None	17 (4.8)	63 (6.2)	26 (4.3)	20 (6.0)	5 (4.5)	3 (4.5)	3 (11.5)	3 (8.3)
Autopsy participants in which residents were in charge								
0 time	344 (98.0)	514 (50.6)	245 (40.3)	120 (35.6)	40 (35.7)	23 (34.3)	8 (30.8)	13 (36.1)
1 time	6 (1.7)	498 (49.1)	268 (44.1)	133 (39.5)	39 (34.8)	26 (38.8)	10 (38.5)	7 (19.4)
2 time	1 (0.3)	2 (0.2)	93 (15.3)	59 (17.5)	17 (15.2)	12 (17.9)	4 (15.4)	7 (19.4)
3 time	0 (0)	0 (0)	2 (0.3)	25 (7.4)	9 (8.0)	1 (1.5)	1 (3.9)	4 (11.1)
4 time	0 (0)	1 (0.1)	0 (0)	0 (0)	7 (6.3)	3 (4.5)	0 (0)	2 (5.6)
5 time	0 (0)	0 (0)	0 (0)	0 (0)	0 (0)	2 (3.0)	1 (3.9)	1 (2.8)
6 time	0 (0)	0 (0)	0 (0)	0 (0)	0 (0)	0 (0)	2 (7.7)	0 (0)
>7	0 (0)	0 (0)	0 (0)	0 (0)	0 (0)	0 (0)	0 (0)	2 (5.6)
Having wish to be a pathologist								
Yes	16 (4.5)	11 (1.1)	4 (0.7)	2 (0.6)	2 (1.8)	3 (4.5)	3 (11.5)	6 (16.7)
No	331 (93.8)	981 (96.7)	585 (96.2)	329 (97.6)	107 (95.5)	61 (91.0)	23 (88.5)	28 (77.8)
Unknown	6 (1.7)	23 (2.3)	19 (3.1)	6 (1.8)	3 (2.7)	3 (4.5)	0 (0)	2 (5.6)
CPC participations								
0 time	33 (9.4)	22 (2.2)	17 (2.8)	10 (3.0)	3 (2.7)	1 (1.5)	0 (0)	0 (0)
1 time	87 (24.7)	279 (27.5)	133 (21.9)	57 (17.0)	17 (15.2)	8 (11.9)	4 (15.4)	2 (5.6)
2 time	48 (13.6)	116 (11.4)	110 (18.1)	55 (16.4)	17 (15.2)	4 (6.0)	4 (15.4)	7 (19.4)
3 time	33 (9.4)	131 (12.9)	79 (13.0)	39 (11.6)	11 (9.8)	10 (14.9)	6 (23.1)	4 (11.1)
4 time	39 (11.1)	80 (7.9)	49 (8.1)	26 (7.7)	18 (16.1)	14 (20.9)	1 (3.9)	3 (8.3)
5 time	23 (6.5)	72 (7.1)	33 (5.4)	22 (6.6)	13 (11.6)	10 (14.9)	4 (15.4)	1 (2.8)
6 time	6 (1.7)	28 (2.8)	17 (2.8)	15 (4.5)	5 (4.5)	3 (4.5)	1 (3.9)	1 (2.8)
>7	84 (23.8)	286 (28.2)	170 (28.0)	112 (33.3)	28 (25.0)	17 (25.4)	6 (23.1)	18 (50.0)

Note

Data are expressed as number (percentage), mean ± standard deviation, or median [25 percentile, 75 percentile].

The mixed‐effect model results showed that an increasing number of autopsy experiences were not associated with higher GM‐ITE scores (Table 2). Significant variables associated with a more excellent GM‐ITE score were internal medicine rotation for 11–15 months (score difference, 1.889; 95% confidence interval [CI], 0.726–3.053; *p* = 0.001); handling an average of 0–4 (score difference, 0.795; 95% CI, 0.127–1.463; *p* = 0.020) and more than 15 (score difference, 2.013; 95% CI, 0.784–3.241; *p* = 0.001) inpatients; and 30–60 (score difference, 1.143; 95% CI, 0.464–1.821; *p* = 0.001) and 61–90 (score difference, 2.771; 95% CI, 1.643–3.899; *p* < 0.001) min of study per day.

**TABLE 2:** Estimates of the mixed‐effect models for GM‐ITE score adjusting for the number of autopsies and deaths at each hospital (Model 1) or the ratio of these numbers (Model 2)^a^

	Model 1				Model 2			
	Estimate	95% CI		*p*	Estimate	95% CI		*p*
Hospital‐level variables								
Number of autopsies (per 10)^c^	−0.017	−0.388	0.353	0.928	—	—		—
Number of deaths (per 100)^c^	0.044	−0.158	0.246	0.671	—	—		—
Autopsy/death ratio	—	—		—	2.213	−13.710	18.136	0.785
University Hospital	−1.291	−2.863	0.281	0.107	−1.409	−3.060	0.241	0.094
Urban area	−0.387	−1.068	0.294	0.265	−0.531	−1.242	0.179	0.143
Number of beds	−0.049	−0.309	0.211	0.711	−0.080	−0.267	0.108	0.404
GM department	0.563	−0.086	1.212	0.089	0.597	−0.069	1.263	0.079
Resident‐level variables								
Female	0.243	−0.233	0.719	0.317	0.337	−0.155	0.828	0.180
Internal medicine rotation								
0–5 months		Reference				Reference		
6–10 months	0.847	−0.243	1.937	0.128	1.082	−0.096	2.259	0.072
11–15 months	1.889	0.726	3.053	0.001	2.002	0.750	3.254	0.002
16–20 months	1.378	−0.363	3.119	0.121	1.804	−0.025	3.633	0.053
>21 months	−0.593	−4.037	2.852	0.736	−0.347	−3.813	3.119	0.844
ED duty per month								
0 per month		Reference				Reference		
1–2 per month	1.141	−0.464	2.747	0.163	1.170	−0.543	2.884	0.180
3–5 per month	1.312	−0.197	2.822	0.088	1.357	−0.265	2.979	0.101
>6 per month	1.481	−0.162	3.124	0.077	1.522	−0.227	3.272	0.088
Unknown	1.360	−1.216	3.936	0.301	1.558	−1.105	4.220	0.251
Average number of inpatients in charge								
0–4		Reference				Reference		
5–9	0.795	0.127	1.463	0.020	0.821	0.132	1.511	0.020
10–14	0.668	−0.178	1.514	0.121	0.845	−0.033	1.723	0.059
>15	2.013	0.784	3.241	0.001	1.913	0.602	3.224	0.004
Unknown	−0.521	−1.901	0.858	0.459	−0.620	−2.112	0.873	0.416
Study time per day								
0–30 min		Reference				Reference		
30–60 min	0.535	0.011	1.059	0.045	0.518	−0.025	1.061	0.061
61–90 min	1.143	0.464	1.821	0.001	1.007	0.297	1.717	0.005
>91 min	2.771	1.643	3.899	0.000	2.726	1.533	3.920	0.000
None	−0.435	−1.457	0.588	0.404	−0.183	−1.247	0.880	0.735
Autopsy participations^d^								
None		Reference				Reference		
1 or 2	−0.005	−0.700	0.690	0.989	−0.091	−0.819	0.636	0.805
3 or 4	0.610	−0.234	1.454	0.157	0.466	−0.407	1.340	0.295
5 or 6	0.794	−0.584	2.172	0.259	0.736	−0.660	2.131	0.301
>6	−2.169	−4.206	−0.131	0.037	−2.286	−4.332	−0.240	0.029
Having wish to be a pathologist								
Yes		Reference				Reference		
No	−0.761	−2.470	0.949	0.383	−0.950	−2.698	0.798	0.286
Unknown	−2.904	−5.088	−0.720	0.009	−3.308	−5.581	−1.035	0.004
CPC participations^e^								
None		Reference				Reference		
1 or 2	−0.573	−1.900	0.754	0.397	−0.713	−2.109	0.683	0.317
3 or 4	0.128	−1.246	1.502	0.855	0.095	−1.345	1.535	0.897
5 or 6	0.994	−0.483	2.470	0.187	0.901	−0.638	2.440	0.251
>6	0.726	−0.659	2.111	0.304	0.518	−0.931	1.967	0.483

^a^ Both models adjusted for hospital variation as normal random intercepts.

^b^ Both models adjusted for hospital variation as normal random intercepts.

^c^ Hospital‐level continuous variables were categorized due to their high value.

^d^ "Autopsy participants in which residents were in charge" variables were removed due to a strong correlation with "Autopsy experiences" variables.

^e^ "CPC participation" variables were categorized in the same way as "Autopsy participations" variables.

The results of the log‐linear and linear models showed that the more residents participated in CPCs, the more they experienced autopsies (Table 3). The other resident‐level variables associated with autopsy experiences were study time of 61–90 (mean ratio, 1.22; 95% CI, 1.11–1.34; *p* < 0.0001) and more than 91 (mean ratio, 1.20; 95% CI, 1.02–1.42; *p* < 0.0001) min per day. Residents who wished to be pathologists experienced more autopsies (*p* = 0.005). Hospital‐level variables associated with autopsy experiences were number of autopsies (mean ratio, 1.14; 95% CI, 1.09–1.18; *p* < 0.0001), smaller number of beds (mean ratio, 0.88; 95% CI, 0.85–0.92; *p* < 0.0001), and not working in hospitals in urban areas (95% CI, 0.78–0.97; *p* = 0.013).

**TABLE 3:** Generalized estimating equations for linear and log‐linear models for mean number of autopsy participations among all residents (the number of autopsy experiences was censored at 7)^a^

	Log‐linear model				Linear model			
	Mean ratio^b^	95% CI		*p*	Mean difference^c^	95% CI		*p*
Hospital‐level variables								
Number of autopsies (per 10)	1.14	1.09	1.18	<0.0001	0.22	0.13	0.31	<0.0001
Number of deaths (per 100)	1.00	0.97	1.03	0.873	−0.01	−0.05	0.04	0.742
Number of beds (per 100)	0.88	0.85	0.92	<0.0001	−0.19	−0.25	−0.13	<0.0001
University Hospital	1.05	0.82	1.33	0.701	0.04	−0.37	0.46	0.841
Urban area	0.87	0.78	0.97	0.013	−0.24	−0.43	−0.06	0.009
GM department	0.95	0.86	1.06	0.382	−0.08	−0.27	0.11	0.397
Resident‐level variables								
Female	0.99	0.93	1.06	0.750	−0.03	−0.14	0.09	0.654
Internal medicine rotation								
0–5 months		Ref				Ref		
6–10 months	1.12	0.93	1.36	0.232	0.20	−0.11	0.52	0.198
11–15 months	1.16	0.95	1.42	0.139	0.26	−0.07	0.59	0.120
16–20 months	1.02	0.77	1.35	0.902	0.06	−0.38	0.50	0.786
>21 months	0.91	0.60	1.37	0.640	−0.13	−0.75	0.49	0.679
ED duty per month								
0 per month		Ref				Ref		
1–2 per month	1.04	0.81	1.34	0.747	0.09	−0.34	0.51	0.692
3–5 per month	1.09	0.86	1.38	0.490	0.14	−0.26	0.55	0.483
>6 per month	1.23	0.93	1.62	0.144	0.37	−0.11	0.85	0.129
Unknown	0.92	0.69	1.23	0.574	−0.16	−0.65	0.33	0.523
Average number of inpatients in charge								
0–4		Ref				Ref		
5–9	1.05	0.95	1.16	0.355	0.08	−0.10	0.26	0.371
10–14	1.02	0.90	1.16	0.762	0.04	−0.17	0.25	0.719
>15	0.99	0.86	1.13	0.857	−0.04	−0.26	0.19	0.742
Unknown	1.00	0.84	1.18	0.975	−0.02	−0.31	0.28	0.918
Study time per day								
0–30 min		Ref				Ref		
31–60 min	1.04	0.96	1.12	0.329	0.06	−0.06	0.18	0.341
61–90 min	1.22	1.11	1.34	<0.0001	0.35	0.18	0.52	<0.0001
>91 min	1.20	1.02	1.42	0.030	0.34	0.01	0.68	0.044
None	1.12	0.95	1.31	0.188	0.20	−0.08	0.48	0.169
Having wish to be a pathologist								
Yes		Ref				Ref		
No	0.68	0.52	0.89	0.005	−0.84	−1.50	−0.18	0.012
Unknown	0.81	0.58	1.12	0.206	−0.53	−1.28	0.21	0.162
CPC participations								
None		Ref				Ref		
1	1.17	0.95	1.45	0.141	0.24	−0.05	0.54	0.105
2	1.48	1.19	1.83	0.000	0.62	0.31	0.93	<0.0001
3	1.46	1.16	1.85	0.002	0.60	0.25	0.95	0.001
4	1.52	1.21	1.91	0.000	0.66	0.32	1.00	0.000
5	1.56	1.22	2.00	0.000	0.71	0.32	1.10	0.000
6	1.66	1.27	2.16	0.000	0.84	0.40	1.29	0.000
>6	1.49	1.19	1.87	0.001	0.65	0.32	0.97	0.000

^a^ Estimated by fitting the working generalized linear models with the canonical form (i.e., assuming Poisson distribution in log‐linear model and normal distribution in linear model for outcome). Confidence intervals and *p* values were based on robust standard errors that treated hospitals as clusters, with the independence working correlation. Note that because the highest category of the number of autopsy participation was censored at 7 (outcome values range from 0 to 7), estimates may include the bias from the "ceiling effect."

^b^ Interpretable as increase (>1) or decrease (<1) in average number of autopsy experiences on multiplicative scale.

^c^ Interpretable as increase (>0) or decrease (<0) in average number of autopsy experiences on additive scale.

## 4 DISCUSSION

To our knowledge, this is the first study that investigated the actual autopsy conditions during a 2 year clinical residency in Japan. More than half of the study participants experienced no or only one autopsy per year.^18^ No correlation was observed between the number of autopsy experiences and GM‐ITE score. Based on these two facts, autopsy experience does not significantly affect residents' clinical competence. With the current low autopsy participation, autopsy is not beneficial for improving residents' clinical competence.

Hospital characteristics affecting whether residents' participation in many autopsies were those located in rural areas, with a small number of beds, and performing a large number of autopsies. The former two characteristics, located in rural areas with a small number of beds, are also related to high GM‐ITE scores.^14^, ^17^ Although we could not verify the relationship between autopsies and the clinical knowledge of residents, autopsy experience might be a factor ensuring the quality of clinical residency at rural hospitals.

Resident characteristics associated with participation in more autopsies were longer study time, more CPC experiences, and pathologist aspirants. In addition, longer study time is also related to high GM‐ITE scores in this study. CPCs are where autopsy cases can be examined from pathological and clinical perspectives. Residents attending more CPCs must be highly motivated and manage their patients from a holistic view. This attitude toward patients is the same as the position of the Association of Pathology Chairs, stating that autopsy training in pathology residency should integrate anatomic and clinical laboratory education.^19^ Moreover, this attitude leads to the ideal physicians setting out in the Japanese PGME.^9^

The reported causes for the decrease in autopsy rate worldwide are cause of death determined before death, no financial support to the pathologist for autopsy procedures, fear of medical malpractice, and changes in public awareness toward autopsies.^2^, ^3^, ^7^, ^20^, ^21^ According to the questionnaire survey, 80% of attending physicians in Japanese teaching hospitals were educating about autopsies. Some requested the redefinition of an autopsy.^10^ We found that several highly motivated residents, such as those with more CPC participation, experienced more autopsies. The improvement of a CPC integrating clinical medicine and pathology does increase the number of autopsy participation.^10^ Moreover, an autopsy may support clinical competence if residents can actively participate in their patients' autopsies.

This study has several limitations. First, this study involved a small sample size. Only 2254 of the 8489 PGY‐2 residents in Japan participated in this study.^22^ Their program directors were responsible for the decision to participate in the GM‐ITE. PGY‐2 residents taking the GM‐ITE, who participated in this survey, were analyzed. There might have been a sampling bias in which the highly motivated teaching hospitals might have involved more participants in the GM‐ITE. However, the number of residents who took the GM‐ITE increased, and only 341 residents (11%) who took the GM‐ITE were denied participation in this survey. Second, resident characteristics, such as emergency room duty per month, number of inpatients handled, and number of autopsies experienced, could be influenced by recall or cognitive bias, since it was a questionnaire study. We used the term “on average” in the questionnaire for these items over the 1 year before the test date or the 2 year clinical residency. Third, the number of autopsies performed in each hospital used in this study was from 2017. Although we could not use the 2018 data, we thought that the number of autopsies at each hospital rarely changed.

In conclusion, we reported the current status of autopsy in clinical residency in Japan based on this questionnaire study involving PGY‐2 residents. We found that more than half of the residents experienced no or only one autopsy. GM‐ITE scores were not correlated with the number of autopsy experiences. Further study is required for improving autopsy training during clinical residency.

## CONFLICT OF INTEREST

YN and YT participated on the General Medicine In‐training Examination project committee of the Japan Organization of Advancing Medical Education Program and received a reward.

## Supporting information

Table S1‐S2
